# Endophytic Bacteria From the Roots of the Medicinal Plant *Alkanna tinctoria* Tausch (*Boraginaceae*): Exploration of Plant Growth Promoting Properties and Potential Role in the Production of Plant Secondary Metabolites

**DOI:** 10.3389/fmicb.2021.633488

**Published:** 2021-02-03

**Authors:** Angélique Rat, Henry D. Naranjo, Nikos Krigas, Katerina Grigoriadou, Eleni Maloupa, Alicia Varela Alonso, Carolin Schneider, Vassilios P. Papageorgiou, Andreana N. Assimopoulou, Nikolaos Tsafantakis, Nikolas Fokialakis, Anne Willems

**Affiliations:** ^1^Laboratory of Microbiology, Department Biochemistry and Microbiology, Faculty Sciences, Ghent University, Ghent, Belgium; ^2^Laboratory of Conservation and Evaluation of Native and Floricultural Species, Institute of Plant Breeding and Genetic Resources, Hellenic Agricultural Organization Demeter, Thessaloniki, Greece; ^3^Institut für Pflanzenkultur, Schnega, Germany; ^4^Organic Chemistry Laboratory, School of Chemical Engineering, Aristotle University of Thessaloniki and Center of Interdisciplinary Research and Innovation of AUTh (CIRI-AUTh), Natural Products Research Centre of Excellence (NatPro-AUTH), Thessaloniki, Greece; ^5^Division of Pharmacognosy and Natural Products Chemistry, Department of Pharmacy, National and Kapodistrian University of Athens, Athens, Greece

**Keywords:** endophytes, isolation, *Alkanna tinctoria*, alkannin, shikonin, hairy roots

## Abstract

Alkannin and shikonin (A/S) are enantiomeric naphthoquinones produced in the roots of certain plants from the Boraginaceae family such as *Lithospermum* spp. and *Alkanna* spp. They possess antimicrobial, anti-tumoral and wound healing properties. The production of secondary metabolites by *Alkanna tinctoria* might be influenced by its endomicrobiome. To study the interaction between this medicinal plant and its bacterial endophytes, we isolated bacteria from the roots of wild growing *Alkanna tinctoria* collected near to Athens and Thessaloniki in Greece. Representative strains selected by MALDI-TOF mass spectrometry were identified by partial 16S rRNA gene sequence analysis. In total, 197 distinct phylotypes of endophytic bacteria were detected. The most abundant genera recovered were *Pseudomonas*, *Xanthomonas*, *Variovorax*, *Bacillus*, *Inquilinus*, *Pantoea*, and *Stenotrophomonas*. Several bacteria were then tested *in vitro* for their plant growth promoting activity and the production of cell-wall degrading enzymes. Strains of *Pseudomonas*, *Pantoea*, *Bacillus* and *Inquilinus* showed positive plant growth properties whereas those of Bacteroidetes and *Rhizobiaceae* showed pectinase and cellulase activity *in vitro*. In addition, bacterial responses to alkannin and shikonin were investigated through resistance assays. Gram negative bacteria were found to be resistant to the antimicrobial properties of A/S, whereas the Gram positives were sensitive. A selection of bacteria was then tested for the ability to induce A/S production in hairy roots culture of *A. tinctoria*. Four strains belonging to *Chitinophaga* sp., *Allorhizobium* sp., *Duganella* sp., and *Micromonospora* sp., resulted in significantly more A/S in the hairy roots than the uninoculated control. As these bacteria can produce cell-wall degrading enzymes, we hypothesize that the A/S induction may be related with the plant-bacteria interaction during colonization.

## Introduction

Plants communicate and interact with a wide variety of microorganisms. They release water-soluble sugars, organic acids, ionic compounds, phenolics, hormones, and other metabolites into the rhizosphere, thus providing nutrients to microorganisms. Due to the richness of nutrients in this soil region, compared to bulk soil, the rhizosphere is enriched with microorganisms, leading to increased microbial interactions (Sasse et al., 2018; Yu and Hochholdinger, 2018). Rhizospheric microorganisms influence plant growth through soil nutrient recycling and nutrient uptake. For example, bacteria such as *Acidobacterium* sp., *Pedobacter* sp., *Muciliginibacter* sp., and *Cellulomona*s sp. play an important role in the recycling of plant polysaccharides such as cellulose, pectin and lignin (López-Mondéjar et al., 2016; Poulsen et al., 2016; Belova et al., 2018). Several bacteria including those from the genera *Pseudomonas*, *Pantoea*, and *Bacillus* have the ability to solubilize and therefore make accessible insoluble phosphate to plants (Ghyselinck et al., 2013). Similarly, rhizobia can provide plants with combined nitrogen via biological nitrogen fixation (Mabrouk et al., 2018). Soil microorganisms can also influence plant growth through the secretion of growth hormones such as indole acetic acid (IAA) or ethylene. Indeed, several studies have shown that plant inoculation with IAA-producing bacteria promote plant growth significantly (Patten and Glick, 2002; Mohite, 2013; Chandra et al., 2018). Furthermore, rhizospheric bacteria can play a role in plant defense and metabolite production (Lambers et al., 2009; Berendsen et al., 2012; Huang et al., 2019). They can first compete with pathogens for nutrients and space in the rhizosphere. For instance, siderophore-producing bacteria can contend with soil pathogens for iron uptake and consequently prevent the root colonization by these microorganisms. As iron is essential for the growth of many organisms and is involved in biofilm formation that is needed for successful root colonization, its uptake by beneficial bacteria can prevent pathogen infections (Ramey et al., 2004; Ahmed and Holmström, 2014). Microorganisms can also produce secondary metabolites with antimicrobial properties. For example, *Burkholderia* sp. showed antimicrobial activities against the plant pathogens *Phytophthora capsici*, *Fusarium oxysporum*, and *Rhizoctonia solani* through the production of pyrrolnitrin (Jung et al., 2018). In addition, some microbes can induce plant defenses. Asghari et al. (2020) demonstrated that *Pseudomonas* sp. Sn48 and *Pantoea* sp. Sa14 induce plant production of phytoalexins and polyamines resulting in a reduction of the *Agrobacterium tumefaciens* gall.

Among the microorganisms interacting with plants, endophytes are defined as those that colonize the internal tissues of plants during the entire or part of their host’s lifecycle without causing external damage (Ryan et al., 2008; Anjum and Chandra, 2015; Orlikowska et al., 2017). Like rhizospheric microorganisms, they can promote plant growth and influence plant metabolites. In the case of medicinal plants, endophytes contribute to or are responsible for their host’s pharmaceutical properties (e.g., antioxidant and/or antimicrobial properties; Köberl et al., 2013; Brader et al., 2014; Maggini et al., 2017). The inoculation of *Bacillus subtilis* BERA 71 to chickpea increased the plant biomass and reduced the levels of reactive oxygen species and lipid peroxidation under salinity stress. This effect was associated with an enhancement of the activities of enzymatic and non-enzymatic antioxidants (Abd_Allah et al., 2017). Endophytes can also influence the antimicrobial activity as well as the production of secondary metabolites in medicinal plants. Pandey et al. (2018) showed that endophytic fungi increase the biomass of *Withania somnifera*, resulting in a higher total plant withanolide content. Moreover, the inoculation of endophytic bacteria stimulated the withanolide biosynthesis pathway by upregulating the expression of key genes within this pathway such as *HMGR*, *DXS*, and *DXR* (Pandey et al., 2018). Endophytes of medicinal plants also produce a wide range of bioactive secondary metabolites such as alkaloids, isoprenoids, flavonoids and indoles (Tan and Zou, 2001; Barman and Bhattacharjee, 2020; Monnanda et al., 2020).

Studies on endophytes are indeed invaluable to the promotion of medicinal plants’ therapeutic properties. *Alkanna tinctoria* (family *Boraginaceae*) is a medicinal plant native to countries of the Mediterranean region (Strid, 2016). The plant produces quinones and phenolic compounds and has antioxidant activities. Moreover, compared to other *Alkanna* species, *A. tinctoria* produces high amounts of alkannin/shikonin (A/S) and their derivatives. These secondary metabolites are enantiomeric naphthoquinones produced by root tissues. They are sequestered to form granules in the phospholipid layer and are accumulated in the apoplastic spaces. Thus, they can be found in the cork layer of mature roots and their accumulation leads to a red or purple coloration of the root (Brigham et al., 1999; Singh et al., 2010; Tatsumi et al., 2016). The A/S derivatives are strongly involved in the antimicrobial activity of the plant and form a chemical barrier against soil-borne microorganisms. They are also known for wound healing, anticancer and anti-inflammatory properties (Papageorgiou et al., 1999, 2008) and they comprise the active pharmaceutical ingredients of strong wound healing medicines approved by the National Organization for Medicines in Greece. Because of these interesting traits, *A. tinctoria* was chosen for the production of A/S for medical applications (Papageorgiou et al., 2008; Sengul et al., 2009; Tung et al., 2013).

Despite the medicinal importance of wild-growing *A. tinctoria* and the potential bioactive properties of its associated bacteria, no studies have been performed so far on its endophytic bacteria and their possible interactions with the plant. The aims of this work were therefore (i) to explore the diversity of endophytic bacteria associated with *A. tinctoria*, (ii) to screen the isolates for a number of plant-growth promotion properties and for the production of cell-wall degrading enzymes (iii) to evaluate the effect of the A/S produced by the plant on the associated bacteria, and (iv) to test their effect on plant A/S production in a bioassay.

## Materials and Methods

### Isolation of Root Endophytic Bacteria From Wild-Growing *Alkanna tinctoria* Plants

Three botanical expeditions to collect wild-growing *Alkanna tinctoria* were undertaken. The first one was conducted in December 2017, in Northern Greece (Seich Sou area close to the Theater of the Earth, Thessaloniki, Greece), using a special collection permit obtained by the Institute of Plant Breeding and Genetic Resources, Hellenic Agricultural Organization Demeter (IPBGR, HAO Demeter) which is issued annually by the Greek Ministry of Environment and Energy. Samples from this expedition were used to optimize the isolation conditions. The wild plants collected were taxonomically identified and they were given the International Plant Exchange Network (IPEN) accession number GR-1-BBGK-18,6081 for their long-term *ex situ* conservation (including also asexual and *in vitro* propagation trials) at the premises of IPBGR, HAO Demeter. Two further collections were made in April and May of 2018 in Southern Greece (at the University of Athens, Campus of Zografou, Athens) and Northern Greece (Seich Sou area, Thessaloniki), respectively.

After each expedition, a bacterial isolation campaign was conducted. Three plants from each of the collections were chosen to explore the diversity of culturable bacterial endophytes. The roots were first cleaned with tap water to remove soil particles. Subsequently, they were immersed in a solution of 70% ethanol for 5 min. After rinsing with sterile water, the root was further sterilized with a solution of 1.4% of NaOCl for 20 min. The root was rinsed again and immersed in 2% Na_2_S_2_O_3_ for 10 min to neutralize the effect of bleach. A last rinse with sterile water was performed before testing the efficiency of the sterilization process in order to exclude non-endophytes. To do so, the external surface of the root was imprinted onto a sterile plate containing 869 agar medium (Weyens et al., 2012; Eevers et al., 2015) before incubation at 28°C. Only roots without microbial growth on the imprints were used for the isolation of endophytic bacteria. The root material was crushed in 10 mL of sterile phosphate buffer saline (PBS) and a 10-fold dilution series was prepared. The dilutions 10^–2^–10^–7^ were then plated in triplicate.

To determine the isolation conditions (medium, incubation time and temperature) that would yield the widest bacterial diversity, during the first isolation campaign a wide selection of media were compared. Nine culture media supplemented with 50 mg/L cycloheximide were tested, including the commonly used media R2A (Difco), 1/10 TSA (Oxoid), and ISP2 (Oxoid), as well as 1/10 869 medium (Eevers et al., 2015) which is routinely used for the isolation of endophytes. In addition, 1/10 869 medium supplemented with either an infusion of dried *Symphytum officinale* (family *Boraginaceae*) roots or with allantoin was tested. To prepare the former medium supplement, dried *S. officinale* roots (Bioshop, Gent, Belgium) were infused in boiled water for 10 min (28 g/L). The infusion was then filtered and used to resuspend the dry ingredients of medium 1/10 869. Allantoin was added to medium 1/10 869 at four concentrations (0.32, 1.6, 9, 32 mM). Allantoin is a secondary metabolite found in the root of several members of the *Boraginaceae* family (Eevers et al., 2015; Dresler et al., 2017). In this first isolation campaign, plates were incubated at 28°C for 4 days after which all distinct colony types were picked for purification. Plates of the highest dilutions were incubated for an additional 10 days at 20°C to isolate slow growers.

### Identification of the Isolates

Dereplication and identification of the isolates obtained in the different conditions were performed using MALDI-TOF MS and 16S rRNA gene sequencing. To conduct the MALDI-TOF MS, pure 2nd generation cultures were grown. The proteins were then extracted, spotted in duplicate onto MALDI-TOF target plates and overlaid with matrix solution (10 mg/mL α-cyano-4-hydroxycinnamic acid in acetonitrile-water-trifluoroacetic acid) as previously described (Dumolin et al., 2019). The protein profiles were read with the Bruker Microflex^TM^ LT/SH system. The programs SPeDE (Dumolin et al., 2019) and BioNumerics (Applied Mats) were used to cluster the protein profiles. In SPeDe, each unique spectrum is assigned as a reference and clusters of all similar spectra are generated (Dumolin et al., 2019). Thus, strains from which the spectra clustered together were considered as highly similar and only the reference strains were selected for rRNA 16S gene sequencing (Jain et al., 2018).

Genomic DNA was extracted by alkaline lysis according to the method of Niemann et al. (1997). Amplification of the 16S rRNA gene was performed using primers pA (3′-AGAGTTTGATCCTGGCTCAG-5′, forward) and pH (5′-AAGGAGGTGATCCAGCCGCA-3′, reverse). The PCR products were sequenced by Eurofins Genomics, Mix2seq service, with the BKL1 primer (5′-GTATTACCGCGGCTGCTGGCA-3′, reverse) and the sequences obtained were identified with EzTaxon database (Yoon et al., 2017). We considered two sequences as belonging to the same species when they were at least 98.7% identical and as belonging to the same genus when they were at least 94.8% identical (Moreira and López-García, 2014; Yarza et al., 2014).

### Plant Growth-Promoting Activity of Endophytic Bacteria

To test bacteria *in vitro* for their plant growth promoting activity, four parameters were evaluated: phosphate solubilization, siderophore production, ACC deaminase activity, and indole acetic acid production.

#### Phosphate Solubilization

NBRIP medium (2.5 g/L Ca_3_(PO_4_); 5 g/L MgCl_2_,6H_2_O; 0.25 g/L MgSO_4_,7H_2_O; 0.2 g/L KCl, 0.1 g/L (NH_4_)_2_SO_4_, and 15 g/L agar) with 10 g/L glucose was used for this test (Nautiyal, 1999). The pH of the medium was adjusted to 7 before autoclaving and small Petri dishes were used (diameter 5.5 cm). Dense bacterial suspensions (OD 0.5–0.6) were made in 1 mL of PBS. Then, 10 μL of each suspension was spotted in the middle of each plate. The test was performed in triplicate. The diameter of clear halos around the bacterial growth was measured after 2, 4, 7, and 10 days of incubation at 28°C. The strain R-42086 (*Pseudomonas* sp.) was used as a positive control (Ghyselinck et al., 2013).

#### Siderophore Production

To test the production of siderophores, the CAS (chrome azurol S) blue medium was prepared according to Louden et al. (2011). Small Petri dishes were used (diameter 5.5 cm). Dense bacterial suspensions (OD 0.5–0.6) were made in 1 mL of sterile PBS. Then, 10 μL of each suspension was spotted in the middle of each plate. The test was performed in triplicate. The halo diameter was observed and measured after 2, 4, 7, and 10 days of incubation at 28°C. The strain R-42086 (*Pseudomonas* sp.) was used as a positive control (Ghyselinck et al., 2013).

#### 1-Aminocyclopropane-1-Carboxylate (ACC) Deaminase Activity

DFS medium was prepared for the ACC deaminase test following the protocol from Penrose and Glick (2003). The glucose and the citric acid were filter sterilized and added to the autoclaved medium. To conduct the experiment, the bacteria were first grown in DFS medium + 2 g (NH_4_)_2_SO_4_ for 72 h at 28°C, 100 rpm. Then, in a 96-well plate, for each strain 15 μL of the liquid culture was added in 135 μL of DFS medium + 3 mM ACC. Each strain was tested in triplicate. The DFS medium without any nitrogen source was used as control. As blanks, the outermost wells of the plate were filled with the culture medium free of bacteria. After 48 h at 28°C, the OD was read at 590 nm. Then, 50 μL of the culture was re-transferred to fresh DFS + 0.3 M ACC. This procedure was repeated twice to exhaust any residual stock of nitrogen. The strain R-42058 *(Pseudomonas* sp.) was used as a positive control and the ACC deaminase activity was evaluated by comparison with the blank (Ghyselinck et al., 2013).

#### IAA Production

Yeast malt extract medium (YM, peptone 5 g/L, yeast extract 3 g/L, malt extract 3 g/L, pH 6.2) was used to test for the production of indole acetic acid (IAA) in liquid culture (Apine and Jadhav, 2011; Mohite, 2013). Bacteria were grown in R2B medium (Difco) for 72 h at 28°C, 100 rpm. Then, 1.5 mL of each bacterial culture was centrifuged (20°C, 5 min, 14,000 rpm), the supernatant was removed, and the pellet was resuspended in sterile distilled water. Then, 100 μL of the suspension were inoculated in 900 μL of YM medium with and without tryptophan (1 g/L). The outermost wells of the plate were filled with the culture medium free of bacteria as blanks. The test was performed in triplicate. Incubation was at 28°C for 72 h. The strain R-42086 (*Pseudomonas* sp.) was used as a positive control (Ghyselinck et al., 2013).

After incubation, the plates were centrifuged at 20°C, 3,700 rpm for 5 min. Then, 50 μL of the supernatant was transferred to flat bottom 96-wells plate and 100 μL of Salkowsky’s reagent (2% 0.5 M FeCl_3_ in 35% HClO_4_ solution) was added and mixed by pipetting. The plate was then kept in the dark for 30 min before measuring the optical density. A calibration curve was made with 0, 0.5, 1, 2, 5, 10, 15, 20, 25, 30 μg of IAA/mL.

### Enzymatic Activities

Our isolates’ ability to recycle plant polysaccharides such as cellulose, pectin and lignin was tested through phenotypic enzymatic assays.

#### Pectinase and Cellulase Activities

Depending on the objective of the test, a minimum medium (4 g/L KH_2_PO_4_, 6 g/L Na_2_HPO_4_, 2 g/L (NH_4_)_2_SO_4_, 1 g/L yeast extract, 0.001 g/L FeSO_4_, 0.2 g/L MgSO_4_, 0.001 g/L CaCl_2_, 0.00001 g/L H_3_BO_3_, 0.00001 g/L MnSO_4_, 0.00007 g/L ZnSO_4_, 0.00005 g/L CuSO_4_, 0.00001 g/L MoO_3_, 15 g/L agar, pH 7) supplemented with 5 g/L of either pectin or carboxymethyl cellulose was used for this assay. Dense bacterial suspensions were made in sterile PBS (OD 0.5–0.6). Then, 10 μL of each bacterial suspension was spotted onto the center of the plate. The test was performed in triplicates and plates were incubated at 28°C for 5 days. Plates were then checked for the presence of a solubilization halo. The halo was visualized by the addition of 0.01% congo red and 1% hexadecyltrimethylammonium bromide (CTAB) solutions for the cellulase and pectinase tests, respectively. The strain LMG 16323 (*Cellulomonas cellasea*) was used as a positive control for the cellulase assay whilst strain LMG 2404 (*Pectobacterium carotovorum* subsp. *carotovorum*) was used as a positive control for the pectinase test.

#### Ligninolytic Activity

This assay was conducted in two steps: a first screening on minimum medium (4 g/L KH_2_PO_4_, 6 g/L Na_2_HPO_4_, 2 g/L (NH_4_)_2_SO_4_, 1 g/L yeast extract, 0.001 g/L FeSO_4_, 0.2 g/L MgSO_4_, 0.001 g/L CaCl_2_, 0.00001 g/L H_3_BO_3_, 0.00001 g/L MnSO_4_, 0.00007 g/L ZnSO_4_, 0.0000 5 g/L CuSO_4_, 0.00001 g/L MoO_3_, 15 g/L agar, pH 7) supplemented with 5 g/L lignin and a second test of selected bacteria on the same minimum medium with 25 mg/L of methylene blue.

To screen for ligninolytic activity, dense bacterial suspensions (OD 0.5–0.6) were made in sterile PBS. Then, 10 μL of each bacterial suspension was spotted onto the middle of the plate. The experiment was conducted in triplicates and the plates were incubated at 28°C for 5 days before checking for bacterial growth. Bacteria that were able to grow on lignin medium were transferred two more times to the same medium to ensure that survival was independent of the previous nutrient stock.

After a total of three transfers, strains that retained the ability to grow were selected to check for their ability to solubilize methylene blue. Structurally, methylene blue is very similar to lignin and both compounds can be degraded by the same enzymes. When methylene blue is degraded, it loses its color and can therefore be used to identify lignin-degrading bacteria (Bandounas et al., 2011). Dense bacterial suspensions were made in sterile PBS. Then, 10 μL of each bacterial suspension was spotted onto the middle of the plate. The plates were incubated for 1 week at 28°C and growth was checked daily. Bacteria able to metabolize methylene blue are expected to produce bright halos around their colonies.

### Susceptibility Assay

Susceptibility assays were conducted to assess the sensitivity of *A. tinctoria* endophytic bacteria isolates to the antimicrobial effects of A/S derivatives. The diffusion assay using filter disks described by Bauer et al. (1966) was performed. The concentration tested was 50 μg per filter disk, following Brigham et al. (1999). A mixture of alkannin/shikonin and their derivatives (A/S mixture) was isolated after extraction and further fractionation steps from *A. tinctoria* roots, as described in Assimopoulou et al. (2008) and Tappeiner et al. (2013). The mixture was first solubilized in acetone to a concentration of 46.7 mg/mL and then diluted in sterile water to a concentration of 2.5 mg/mL. Then, 20 μL of each test solution were used to inoculate the filter disks. In total, six filter disks were used in this assay: three were inoculated with the A/S mixture (50 μg/filter disk), one with acetone-water solution (5.9% acetone; used as an A/S mixture solvent control), one with ampicillin (50 μg/filter disk; as a control antibiotic), and one with MilliQ water (as ampicillin solvent control). Dense bacterial suspensions were prepared in PBS and 100 μL of each suspension was inoculated by spreading on R2A plates. Individual filter disks containing 20 μL of each test solution were left to dry for 30 min before being transferred onto the plates. The test was performed in triplicate. Control strains were selected based on the results reported by Brigham et al. (1999): LMG 8224 *(Staphylococcus aureus* subsp. *aureus*) and LMG 7135 (*Bacillus subtilis* subsp. *subtilis*) as sensitive strains and LMG 8223 (*Escherichia coli*) as a resistant strain.

### Induction of A/S Production in Hairy Roots of *A. tinctoria*

Hairy roots of *A. tinctoria* were produced with a modified protocol (Brigham et al., 1999; Fukui et al., 1999; Kim et al., 2009) as follows: *Agrobacterium rhizogenes* strain LMG 149 was grown in 40 mL Nutrient Broth (Difco) medium at 28°C for 48 h in the dark and at 100 rpm agitation. The leaves of sterile *A. tinctoria* explants provided by IPBGR, HAO Demeter (Thessaloniki, Greece) were cut and inoculated at different spots with a syringe filled with a bacterial solution of *A. rhizogenes* (OD = 0.5–0.6). Each inoculated leaf was transferred onto solid Murashige and Skoog (MS, Duchefa) medium supplemented with 3% sucrose and 0.8% phytoagar (Duchefa) and kept for 1 week at 25°C in the dark until roots appeared. As a control, non-inoculated leaves were cultivated under the same conditions. Developing roots (approximately 1 cm in length), were cut off and transferred weekly (for a period of 3–4 weeks) to a new MS solid medium containing 1% sucrose, 0.8% phytoagar, and 500 mg/L of cefotaxime. The roots were then propagated in liquid MS medium supplemented with 3% sucrose (Brigham et al., 1999; Fukui et al., 1999; Kim et al., 2009).

To evaluate the ability of bacteria to induce A/S production in the hairy roots of *A. tinctoria*, a first screening was performed in 6-well plates. The bacteria for this test were selected based either on their plant growth promoting properties or their enzymatic activities. Firstly, 7 mL of MS + 1% sucrose medium were added to each well, followed by the aseptic addition of young root segments. Bacteria were inoculated to a final OD of 0.001/mL. One 6-well plate was prepared per strain. In addition, an uninoculated control plate was prepared. Plates were incubated in the dark at 100 rpm agitation at 25°C for 1 week. The roots from individual wells were pooled per plate and lyophilized. The extraction of A/S was conducted overnight with 8 mg of dried roots in 250 μL of methanol and the OD was read at 520 nm (Assimopoulou et al., 2009).

Among seven candidates showing an OD > 0.2 at 520 nm in the first screening, four were subsequently tested in a larger volume. Five 100 mL Erlenmeyer filled with 50 mL of MS + 1% sucrose medium were used per treatment. Pieces of young roots were then carefully added in each Erlenmeyer and incubated for 1 week at 25°C (100 rpm). The Erlenmeyers were then randomized before being inoculated with bacteria to a final OD of 0.001. One uninoculated control (5 replicates) was also prepared. Erlenmeyers were incubated for 1 week at 25°C (100 rpm). The roots from each Erlenmeyer were collected and lyophilized. The extraction was conducted overnight using 40 mg of dried roots in 1,250 μL of methanol. The OD was read at 520 nm and one-factor Anova followed by a Neuman-Keuls test was conducted on the results with the software R x64 3.1.0.

## Results

### Isolation and Identification of Root Endophytic Bacteria

For our study nine different populations of endogenous *Alkanna tinctoria* from Northern and Southern Greece have been explored. The first isolation campaign assessed the media that would allow the growth of a wide diversity of culturable bacteria and yielded 1,483 isolates. MALDI-TOF mass spectra of these isolates were compared and grouped, resulting in 914 representative strains that were selected for genotypic identification by 16S rRNA gene sequencing. This allowed identification of 142 distinct phylotypes of endophytic bacteria following the classification threshold values at genus or species level introduced by Moreira and López-García (2014) and Yarza et al. (2014). The number of isolates and phylotypes recovered for each plant sample and medium is available in Supplementary Table S1. The coverage of bacterial diversity for each medium tested was obtained by dividing the number of unique phylotypes from the selected medium by the total number of phylotypes. The media that yielded the highest diversity were medium 1/10 869 supplemented with 32 mM allantoin and medium 1/10 TSA (Table 1). Moreover, media R2A, 1/10 869 and 1/10 869 supplemented with 0.32 mM allantoin allowed the cultivation of members of additional genera including *Acidovorax*, *Micromonospora*, *Kocuria*, *Diaminobutyricimonas*, and *Muciliginibacter* (Supplementary Figure S1).

**TABLE 1:** Coverage of bacterial diversity of different media, where the number of unique phylotypes in the selected medium wasdivided by the total number of detected phylotypes is given inpercentage (%).

Medium	Coverage of the diversity in %
1/10 869	30.3
1/10 869 + 0.32 mM allantoin	32.6
1/10 869 + 1.6 mM allantoin	28,0
1/10 869 + 9 mM allantoin	31.8
1/10 869 + 32 mM allantoin	47.0
1/10 869 + Plant extract	8.3
ISP2	22.7
1/10 TSA	40.2
R2A	35.6

For the isolation from the second and third collection campaigns, the media R2A, 1/10 TSA, 1/10 869 and 1/10 869 + 32 mM allantoin were used to maximize the culturable diversity. Moreover, the first isolation campaign indicated that extended incubation at 20°C led to a 15.2% increase in diversity at higher dilutions. Most of these isolates were slow-growing Gram-positive bacteria including *Mycobacterium* sp., *Micromonospora* sp., *Lysinimonas* sp., and *Micrococcus* sp. Then in further isolations, the plates were incubated at 20°C for 2 weeks and colonies were picked after 1 and 2 week(s), respectively.

**FIGURE 1:**
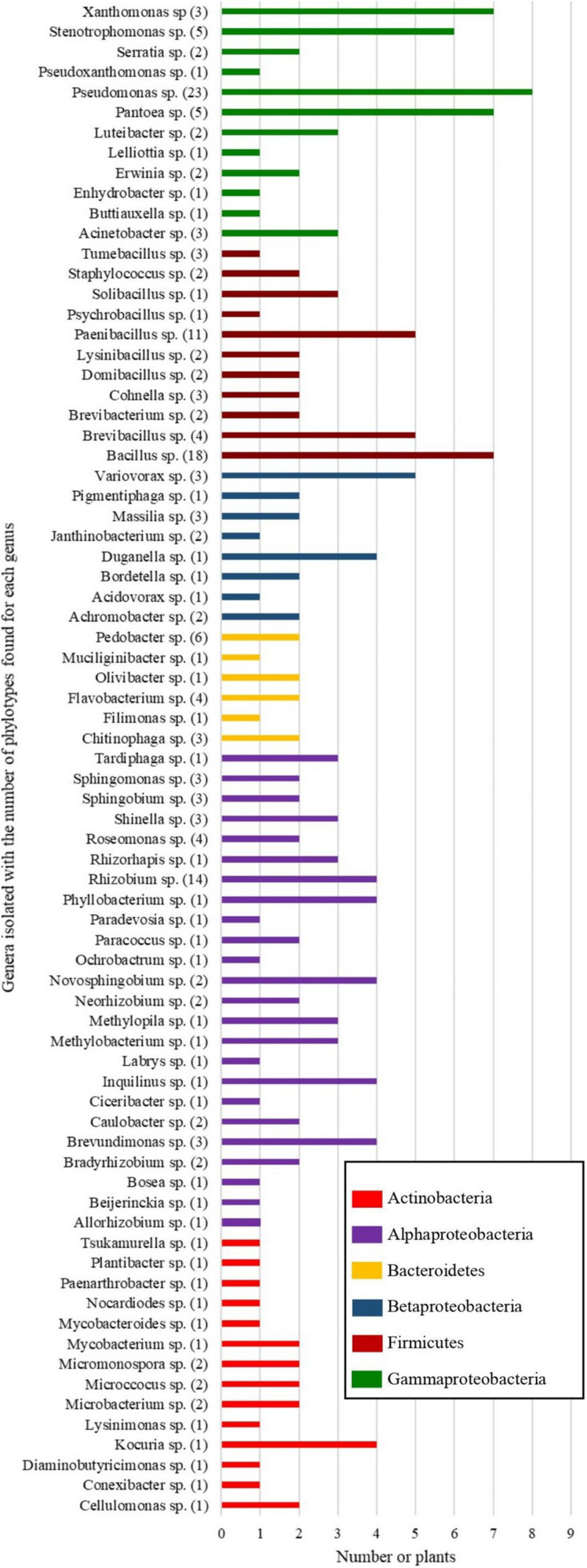
Diversity of genera isolated from wild-growing *Alkanna tinctoria* plants and the number of plant individuals in which they were found. The number of species found per genus is specified in parentheses. Different colors correspond to specific bacterial phyla or classes.

The second and third isolation campaigns using four media only yielded 770 and 582 isolates, respectively. Together with the isolates from the first campaign, this gave 2835 primary bacterial isolates in total. Protein profiles of all these isolates were compared by MALDI-TOF MS and grouped. This allowed the selection of 1,428 representative strains, 914 of which had already been identified by 16S rRNA gene sequencing in the first isolation campaign. The remaining 514 representative strains were also identified by 16S rRNA gene sequencing. In total, among the 1,428 selected representative strains, 197 unique phylotypes were recovered, belonging to 14 genera of Actinobacteria, 6 genera of Bacteroidetes, 11 genera of Firmicutes, and 44 genera of Proteobacteria. Also, three potentially new genera and 40 potentially new species were recovered from the roots of wild *A. tinctoria* plants. The bacteria were distributed in four phyla, i.e., Bacteroidetes, Proteobacteria, Actinobacteria, and Firmicutes. The plants of the third isolation campaign yielded relatively more Gram positive bacteria (Actinobacteria and Firmicutes) compared to the other plants. Similarly, the plants of the 1st and 2nd isolation campaigns yielded more Alphaproteobacteria and Gammaproteobacteria, respectively (Supplementary Figure S2). Most of the isolates belonged to the Proteobacteria, particularly the Gammaproteobacteria, Alphaproteobacteria, and Betaproteobacteria. Members of the genera *Pseudomonas* were found in eight of the nine plants studied, whereas *Pantoea, Xanthomonas* and *Bacillus* were recovered from seven *A. tinctoria* plants. Additionally, representatives of *Stenotrophomonas* were found in six of the nine plants (Figure 1). These genera were also isolated from all the tested media (1/10 869, 1/10 869 + 32 mM allantoin, 1/10 TSA and R2A). The isolates that were less than 94.8% similar to the type strain of any bacterial species represent four potentially new genera which are related to *Filimonas*, *Bordetella*, *Cohnella* and *Paenibacillus*. Moreover, 40 bacterial strains that were less than 98.7% similar to the type strain of any bacterial species probably represent potential new species. Of these, members of the Bacteroidetes are related to *Chitinophaga*, *Flavobacterium*, *Mucilaginibacter*, and *Pedobacter*. Potentially new species within the Phylum Firmicutes are related to *Bacillus*, *Cohnella*, *Domibacillus*, *Paenibacillus*, and *Tumebacillus*. Potentially new Proteobacteria isolates are related to *Acinetobacter*, *Allorhizobium*, *Beijerinckia*, *Bradyrhizobium*, *Brevundimonas*, *Janthinobacterium*, *Massilia*, *Ochrobactrum*, *Pseudomonas*, *Roseomonas*, *Rhizobium*, *Rhizorhapis*, *Shinella*, *Sphingobium*, *Sphingomonas*, and *Stenotrophomonas*. Finally, potentially new isolated Actinobacteria members are related to the genera *Cellulomas*, *Conexibacter*, *Diaminobutyricimonas*, *Lysinimonas*, *Mycobacterium*, and *Nocardioide*s.

### Plant Growth-Promoting Activity

**FIGURE 2:**
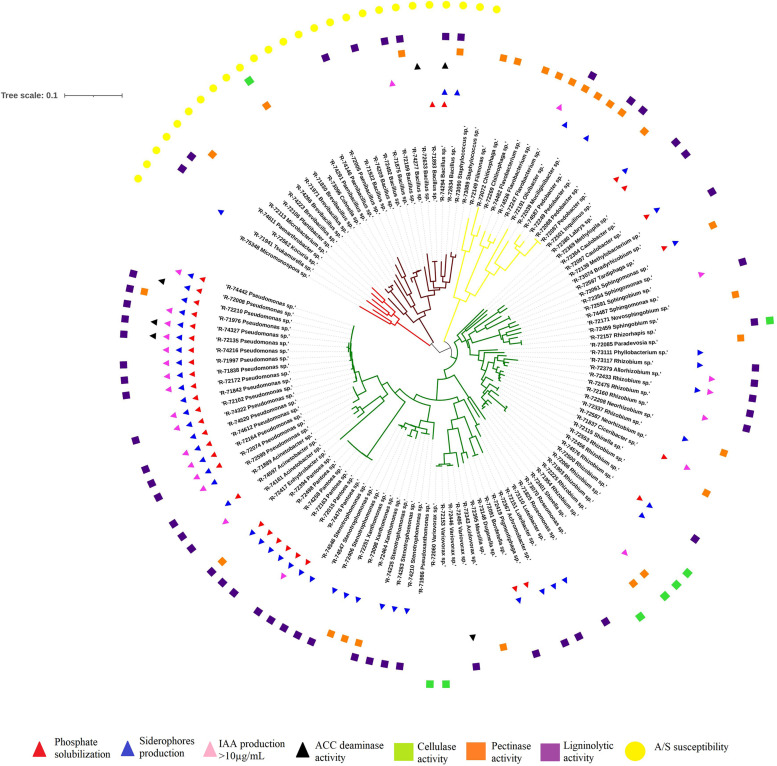
Neighbor-joining phylogenetic tree based on 16S rRNA gene sequences, with Juke-Cantor model, showing the phylogenetic relationships of strains tested *in vitro* for plant growth, cell-wall degrading enzymes and susceptibility to the mixture of A/S. Activities detected are shown in the outer circle. Sequences were aligned using 361 CLC Main Workbench 7.9.1 (Qiagen). Subsequently, a phylogenetic maximum-likelihood tree (1000 bootstraps) was reconstructed and visualized using the iTOL software 5.6.3 (Letunic and Bork, 2019).

One hundred and twenty-seven strains which represented most of the bacterial diversity recovered, were tested for their plant growth promoting activity *in vitro*. The results are summarized in Figure 2 (additional information is provided in the Supplementary Table S2). The strains expressing positive activity for all four tested parameters belonged to the genera *Pseudomonas* and *Bacillus*. The ones expressing positive activity for at least three of the parameters tested belonged to the genera *Pseudomonas*, *Pantoea*, *Inquilinus*, *Rhizobium*, and *Bacillus*. On the other hand, bacteria from the genus *Stenotrophomonas* seem to only be able to produce siderophores. The partial 16S sequences of these 127 tested strains are available in GenBank database under the accession numbers MW353471–MW353597 (Supplementary Table S3).

### Enzymatic Activities

Enzymatic activities of the 127 strains are presented in Figure 2 (additional information is provided in Supplementary Table S2). The cellulase activity seemed to be predominant in the *Rhizobiaceae* family, whereas pectinase activity was abundant among the Bacteroidetes phylum. Among the strains tested, some showed two degrading activities: pectinase and ligninolytic activities were detected for R-72191 (*Olivibacter* sp.), R-72210 (*Pseudomonas* sp.), R-72249 (*Pedobacter* sp.), R-72394 (*Pantoea* sp.), R-72464 (*Xanthomonas* sp.), R-7264, and R-74277 (*Bacillus* sp.); cellulase and pectinase activities for the strain R-71830 (*Brevibacillus* sp.) and cellulase and ligninolytic activities for R-72157 (*Rhizorhapis* sp.).

### Susceptibility Assay

**FIGURE 3:**
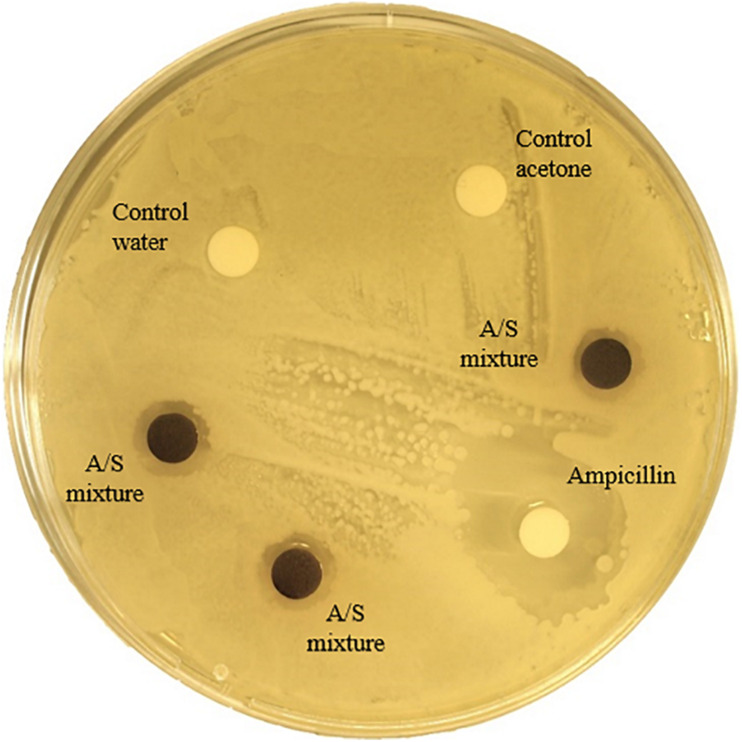
Susceptibility of strain R-72492 (*Bacillus* sp.) to the mixture of alkannin/shikonin derivatives isolated from *Alkanna tinctoria* roots.

Susceptibility to the antimicrobial activity of alkannin and shikonin derivatives (in the form of a mixture of A/S pigments) was studied for the 127 strains tested for PGP and enzymatic activities. A strain was considered as sensitive to A/S derivatives when an inhibition zone of more than 1 mm was observed. An example of the assay is shown in Figure 3. A pattern could be observed in the susceptibility to the A/S mixture (50 μg): all Gram-positive bacteria tested were sensitive whereas the Gram-negative bacteria tested were resistant (Figure 2). No such pattern was observed for ampicillin which was used as a control. As shown in Figure 3, the halos observed as a result of sensitivity to A/S derivatives were always smaller in diameter than those produced as a result of ampicillin sensitivity.

### Induction of A/S Derivatives in Hairy Roots of *A. tinctoria*

In the first screening, treatments with the following bacteria resulted in hairy root extracts showing an OD higher than the control: R-71971 (*Brevibacillus* sp.), R-72060 (*Variovorax* sp.), R-72149 (*Filimonas* sp.), R-72379 (*Allorhizobium* sp.), R-72395 (*Duganella* sp.), R-72401 (*Shinella* sp.), R-72406 (*Stenotrophomonas* sp.), R-73072 (*Chitinophaga* sp.), R-74161 (*Acinetobacter* sp.), and R-75348 (*Micromonospora* sp.) (Table 2). The seven bacteria resulting in an OD > 0.20 were repeated in five replicates in a second assay. The one-factor ANOVA conducted on the results of the second assay showed that the *p*-value = 0.00179 was lower than 0.05. The bacterial treatment had an influence on the A/S content. The comparison made with the test of Newman Keuls are presented Table 3. *Chitinophaga* sp. strain R-73072, *Allorhizobium* sp. strain R-72379, *Massilia* sp. strain R-72395, and *Micromonospora* sp. strain R-75348 induced significantly more A/S derivatives in the hairy roots of *A. tinctoria* when compared to the uninoculated control.

**TABLE 2:** Optical density at 520 nm reflecting the total A/S content of hairy roots extracts of *Alkanna tinctoria* treated with selected bacteria.

Strain	Identification	OD 520 nm	Strain	Identification	OD 520 nm
Control		0.175	R-72269	*Chitinophaga* sp.	0.166
R-71838	*Pseudomonas* sp.	0.124	R-72367	*Achromobacter* sp.	0.158
R-71842	*Pseudomonas* sp.	0.167	R-72379	*Allorhizobium* sp.	**0.222**
R-71875	*Bacillus* sp.	0.163	R-72395	*Massilia* sp.	**0.201**
R-71922	*Bacillus* sp.	0.160	R-72401	*Shinella* sp.	**0.192**
R-71971.	*Brevibacillus* sp.	**0.244**	R-72406	*Stenotrophomonas* sp.	**0.189**
R-72039	*Muciliginibacter* sp.	0.149	R-72433	*Rhizobium* sp.	0.170
R-72060	*Variovorax* sp.	**0.207**	R-72495	*Variovorax* sp.	0.172
R-72074	*Pseudomonas* sp.	0.138	R-72498	*Pantoea* sp.	0.115
R-72097	*Caulobacter* sp.	0.163	R-72501	*Inquilinus* sp.	0.146
R-72135	*Pseudomonas* sp.	0.132	R-73072	*Chitinophaga* sp.	**0.200**
R-72149	*Filimonas* sp.	**0.180**	R-73074	*Bradyrhizobium* sp.	0.170
R-72160	*Rhizobium* sp.	0.162	R-73098	*Xanthomonas* sp.	0.129
R-72191	*Olivibacter* sp.	0.167	R-73110	*Luteibacter* sp.	0.124
R-72210	*Pseudomonas* sp.	0.157	R-74161	*Acinetobacter* sp.	**0.223**
R-72249	*Pedobacter* sp.	0.153	R-74611	*Paenarthrobacter* sp.	0.162
R-72251	*Xanthomonas* sp.	0.142	R-75348	*Micromonospora* sp.	**0.231**

*The values in bold are higher than the OD value of the uninoculated control.*

**TABLE 3:** Comparisons of the OD 520 nm reflecting the total A/S content of hairy roots extracts of *Alkanna tinctoria* inoculated with the best bacterial candidates with Neuman Keuls test.

Strain	Identification	OD 520 nm
Control		0.075 b
R-73072	*Chitinophaga* sp.	**0.120 a**
R-72379	*Allorhizobium* sp.	**0.113 a**
R-75348	*Micromonospora* sp.	**0.108 a**
R-72395	*Massilia* sp.	**0.106 a**
R-72060	*Variovorax* sp.	0.097 ab
R-74161	*Acinetobacter* sp.	0.092 ab
R-71971	*Brevibacillus* sp.	0.090 ab

*Significant differences are highlighted in bold with different letters.*

## Discussion

The cultivation of members of *Boraginaceae* with strong medicinal value such as *A. tinctoria* and *Lithospermum* spp. has proven to be difficult. For example, *Lithospermum* spp. germinate poorly and seedlings are highly susceptible to viral infections. Additionally, plants can only be harvested 2–3 years after germination and the in-field maintenance of these plants has also proved to be difficult (Yazaki, 2017). Applying suitable endophytes to facilitate plant growth by increasing the plant biomass and/or by inducing A/S production can be a sustainable approach toward increasing plant yield and production of these valuable compounds.

As a first step toward the elucidation of the potential role of root endophytic bacteria in the production for secondary metabolites such as A/S derivatives of *A. tinctoria*, we isolated and characterized culturable endophytic bacteria from *A. tinctoria* roots. In total, 197 unique phylotypes were recovered, representing 4 phyla. Also, three potentially new genera and 40 potentially new species were recovered from the roots of wild *A. tinctoria* plants. Most studies to date involving the isolation of endophytic bacteria typically use one type of isolation medium to cultivate a wide range of microorganisms (e.g., nutrient agar or TSA), a set temperature of 28°C and an incubation time between 48 and 72 h (Germida et al., 1998; Mbai et al., 2013; Anjum and Chandra, 2015; Saini et al., 2015). In this study, we showed that a higher diversity of bacteria can be obtained when more than one medium is used. Nutrient-poor media such as R2A or diluted TSA seem better at capturing more diversity than a rich medium such as ISP2 (Table 1 and Supplementary Figure S1). The latter is a medium rich in nitrogen and carbon sources and has been designed for the routine cultivation of *Streptomyces* sp. (Shirling and Gottlieb, 1966). However, as it may favor the fast-growing bacteria, it may be less suitable to isolate slow growing ones. R2A medium was designed for slow-growing bacteria (Reasoner and Geldreich, 1985) and medium 1/10 869 for endophytes (Eevers et al., 2015), whereas TSA medium is non-selective and allows the growth of a wide range of microorganisms (Leavitt et al., 1958). The dilution of TSA increased the growth of slow-growing bacteria when compared to the non-diluted medium. Changes in the quantity and the quality of the carbon source and the nature of electrons donors (e.g., succinate, butyrate, acetate) and acceptors (HEPES solution; Köpke et al., 2005), as well as the addition of supplements like enzymes or antibiotics to isolation media are known to generate higher microbial diversity (Alain and Querellou, 2009). For example, the nitrogen sources of TSA medium are casein and peptones whereas R2A and 869 media contain yeast extract. Moreover, TSA does not have additional carbon sources such as glucose. Poor media limit the risk that fast-growing bacteria eliminate those that grow slower by outcompeting them for nutrients or by negatively affecting the growth conditions (pH or the production of metabolites). Critically, slow-growers or species that grow poorly *in vitro* may be naturally abundant in planta (Alain and Querellou, 2009; Kram and Finkel, 2015). Lower temperature and longer incubation time were also more suited to recovering gram-positive bacteria such as Actinobacteria. Indeed, studies focusing on Actinobacteria demonstrated that an incubation period of 3–4 weeks was necessary for their isolation (Araújo et al., 2000; Coombs and Franco, 2003; Misk and Franco, 2011).

While the isolation conditions are important determining factors for the recovery of bacterial isolates, the diversity observed here between different roots (Figure 1 and Supplementary Figure S2) indicates that other factors are also important. For example, no Gammaproteobacteria such as *Pseudomonas* sp., *Xanthomonas* sp., or *Pantoea* sp. seemed to be present in one of the root samples (Figure 1). Bacterial isolation results can be biased by the origin and storage conditions of the biological sample or the colony picking method, in addition to the isolation conditions, which may have contributed to the variation in phylotype distribution observed between roots and isolation campaigns (Supplementary Figure S2). We studied wild-collected plants from different locations and therefore, parameters such as the genetics of the plant (Haney et al., 2015), local soil composition (Liu et al., 2019), or the presence of neighboring plants (Schlatter et al., 2015; Essarioui et al., 2016) might also influence the diversity of bacteria recovered. Microbiome analysis as well as the study of the growth conditions of the plant in its natural environment might shed light on bacterial variation between samples.

The endophytic bacteria found in most of the root samples were tested for a number of plant-growth promotion (PGP) characteristics. The most interesting species for plant growth promoting activity belong to the genera *Pseudomonas*, *Pantoea*, *Inquilinu*s, *Rhizobium*, and *Bacillus* (Figure 2, Supplementary Table S2). Members of these genera were recovered on all media used for isolation. The ability to grow easily *in vitro* is promising for their application as plant growth promoting rhizobacteria (PGPRs). Some bacteria showed variable results were obtained from the ACC deaminase test (Supplementary Table S2). These bacteria were colored and/or were prone to form biofilms or clumps which can lead inaccurate spectrophotometric readings. To overcome this issue, quantitative analysis might be applied by measuring the amount of α-ketobutyrate at 540 nm (Penrose and Glick, 2003).

Moreover, several bacteria produced *in vitro* cell-wall degrading enzymes such as cellulases, pectinases and ligninases. These properties facilitate bacterial entry and spread within the plant tissues and may contribute to the endophytic lifestyle of these bacteria. Strains belonging to *Pseudomonas* sp., *Pantoea* sp., *Rhizobium* sp., and *Bacillus* sp. expressing PGP and enzymatic activities (Figure 2 and Supplementary Table S2) might thus show stronger impact on plant growth or A/S induction due to their ability to actively colonize the plant.

The resistance assay demonstrated that Gram-negative bacteria are resistant to the antimicrobial properties of A/S derivatives (Figure 2 and Supplementary Table S2). Although the activity of A/S derivatives at 50 μg seemed weak, the experiment confirmed the antimicrobial properties of these naphthoquinones. The antimicrobial activity of A/S has been linked to their ability to form semiquinone radicals by interacting with reactive oxygen species. They can generate endogenous superoxide anion radicals, resulting in their cytotoxicity (Papageorgiou et al., 2008, 1999; Ordoudi et al., 2011). The peptidoglycan cell wall of the Gram-positive bacteria is permeable to molecules with molecular weights in the range of 30,000–57,000 Da, and hence allows for the entry of many small antimicrobials which may partially explain their sensitivity (Lambert, 2002). On the other hand, several mechanisms might be involved in the resistance of the Gram-negative bacteria to A/S, especially through redox processes. Chemical and transcriptomic analyses may provide a better understanding of the processes involved. Most of the bacteria having a positive effect in the *in vitro* plant growth promoting activities tested were Gram-negative and thus resistant to A/S derivatives. This resistance may provide a competitive advantage for colonizing the plant and living in the root tissues. Consequently, the plant may select for plant growth promoting bacteria through the production of A/S derivatives. Alternatively, sensitive bacteria may colonize the plant shortly after germination when the A/S derivatives content is low or they may be present in the seed and are vertically transmitted (Truyens et al., 2014). In the future, these hypotheses might be tested by inoculating isolates that are sensitive to A/S at different plant growth stages and by tracking their presence and abundance within the plant, thus establishing whether or not these strains can colonize and survive in the plant or not.

Studies have shown that in the hairy roots of *A. tinctoria*, A/S derivatives are produced by root border cells of the growing root tips. They are then sequestered as lipid granules in the phospholipid layer of these cells and accumulate in apoplastic spaces. However, how the A/S derivatives are secreted into the environment still remains unclear. Nevertheless, it is known that this process results from plant stress and involves the actin filaments (Brigham et al., 1999; Tatsumi et al., 2016). Depending on where the bacteria colonize the plant, it is possible that they do not come into contact with A/S derivatives. Colonization assays can provide insights into the relationship between plant individuals and bacteria in the presence of alkannins and shikonins. Moreover, it has been shown that ethylene regulates the colonization of plant tissue by bacteria: the absence of ethylene in a plant or the addition of an ethylene inhibitor leads to a higher degree of colonization. Bacteria that are able to affect the ethylene level are more competent at colonizing plants (Hardoim et al., 2008; Liu et al., 2017). The modulation of plant ethylene levels by bacteria can occur by cleavage of 1-aminocyclopropane-1-carboxylate (ACC), a precursor of ethylene or by inhibiting ACC synthase and/or β-cystathionase, both being enzymes involved in the ethylene biosynthesis pathway (Hardoim et al., 2008; Liu et al., 2017). To confirm whether bacteria can effectively colonize and positively affect the plant growth and the total A/S content, in planta tests must be performed.

*Chitinophaga* sp. strain R-73072, *Allorhizobium* sp. strain R-72379, *Massilia* sp. strain R-72395, and *Micromonospora* sp. strain R-75348 showed the ability to directly induce A/S production in *A. tinctoria* hairy root cultures. Hairy roots are genetically modified plant material (Gutierrez-Valdes et al., 2020). The use of MS + 1% sucrose medium, which is a poor medium for bacterial culture, as well as the presence of agitation, might affect the ability of the bacteria to colonize the roots or their abilities to produce biofilm and/or metabolites. During plant colonization, the production of bacterial enzymes or metabolites may induce the production of A/S derivatives by the plant. Our hairy root system served as a general screening tool and may be not optimized for specific plant-bacteria interactions. It might therefore underestimate the number of A/S inducers. Nonetheless, *in vitro* whole plant systems usually employ growth medium similar to MS (Phillips and Garda, 2019) and therefore bacteria showing inducing properties in hairy roots can likely induce A/S *in vitro* plant system. The bacterial strains R-73072, R-72379, and R-72395 inducing A/S in our system, expressed cell-wall degrading enzymatic activities (pectinase or ligninase) *in vitro*. Competent endophytes are able to release cell wall degrading enzymes such as cellulases, xylanases, pectinases, and endoglucanases, which facilitate bacterial entry and spread within the plant tissues (Kandel et al., 2017; Pinski et al., 2019). Nonetheless, colonization remains an invasive process and thus might induce plant defense response, resulting in the synthesis of plant antimicrobials. Alkannin and shikonin derivatives show antimicrobial activities and can thus be considered as plant defense compounds.

Although R-75348 did not show any cell-wall degrading activities *in vitro*, several studies have demonstrated that *Micromonospora* species do have a role in the breakdown of plant cell walls through the production of hydrolytic enzymes (Hirsch and Valdés, 2010; Trujillo et al., 2015). Our test conditions may not have been suitable for this strain. Alternatively, genome analysis might reveal the presence of such enzymes. Moreover, *Micromonospora* sp. is also known to produce antibiotics (Boumehira et al., 2016). Inducing the production of A/S derivatives may depend on plant-bacteria communication through such metabolites. Indeed, some molecules are described as antibiotics because of their effect on microorganisms under *in vitro* conditions although their function in the natural habitat can be different. Such molecules can play a role in plant-microbe interactions but can also act as Microbe-Associated Molecular Patterns (MAMPs). MAMPs are resistance-inducing stimuli recognized by specific plant receptors (Köhl et al., 2019). They can also induce pathways involved in plant defense responses.

We hypothesize that the endophyte’s production of cell-wall degrading enzymes and/or secondary metabolites during plant colonization may cause a plant stress resulting in the production of alkannin and shikonin derivatives.

This study demonstrated the importance of the isolation conditions for the diversity of endophytic isolates recovered from plant roots of *A. tinctoria*. Nutrient-poor isolation media coupled with an incubation temperature of 20°C and an incubation period of a minimum of 2 weeks allowed for a recovery of a high diversity of culturable endophytic bacteria from *A. tinctoria*. Although these findings should be validated in greenhouse or field conditions, the positive *in vitro* results found regarding potential plant growth promotion features suggest that some of these bacteria might be valuable for future in planta applications. These can be of agronomical interest to boost biomass production or crop yield. Such endophytes also have the potential to increase the yield of secondary metabolites such as A/S derivatives from these medicinal plants by increasing the total biomass. As demonstrated in our study, certain endophytes can also induce A/S production in the roots, probably through to the recognition of bacteria by the plant. These findings open-up the perspective of using a combination of endophytes with the potential for plant growth promotion and induction of secondary metabolite production as a sustainable approach toward increasing the production of secondary metabolites in selected medicinal plants.

## Data Availability Statement

The datasets presented in this study can be found in online repositories. The names of the repository/repositories and accession number(s) can be found in the article/Supplementary Material.

## Author Contributions

AW, CS, AAs, and NF designed the study and secured the funding. AR, HN, and AAl designed and performed the experiments. AR analyzed the data and wrote the manuscript. All authors edited, proofread, and approved the manuscript.

## Conflict of Interest

The authors declare that the research was conducted in the absence of any commercial or financial relationships that could be construed as a potential conflict of interest.
