# Supplementary figures and images for: Endophytic Bacteria From the Roots of the Medicinal Plant Alkanna tinctoria Tausch (Boraginaceae): Exploration of Plant Growth Promoting Properties and Potential Role in the Production of Plant Secondary Metabolites
Source: Front Microbiol. 2021 Feb 3;12:633488. doi: 10.3389/fmicb.2021.633488 (PMC7901983; doi:10.3389/fmicb.2021.633488)

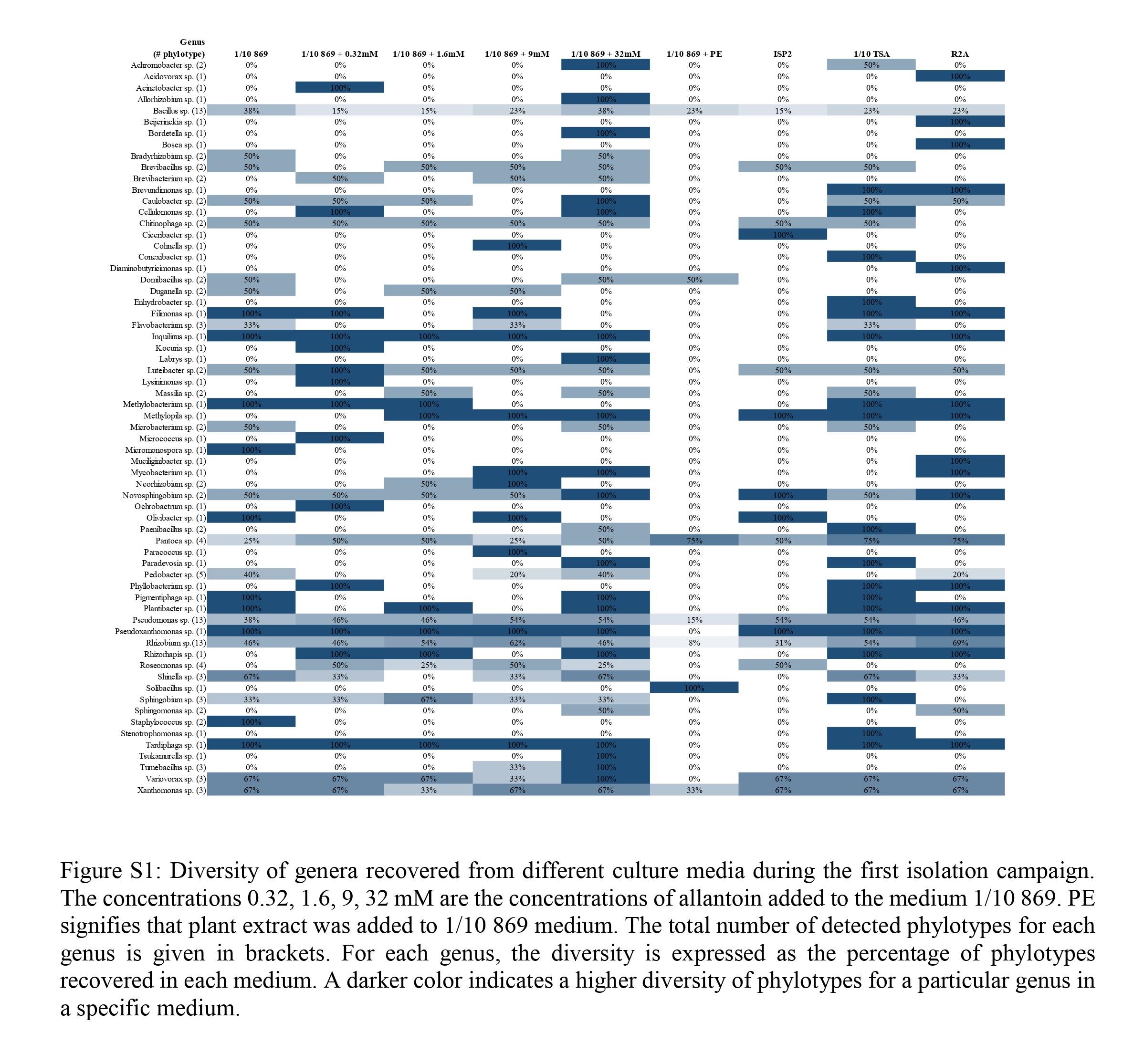

Supplement: Supplementary file 1 [file Image_1.JPEG]

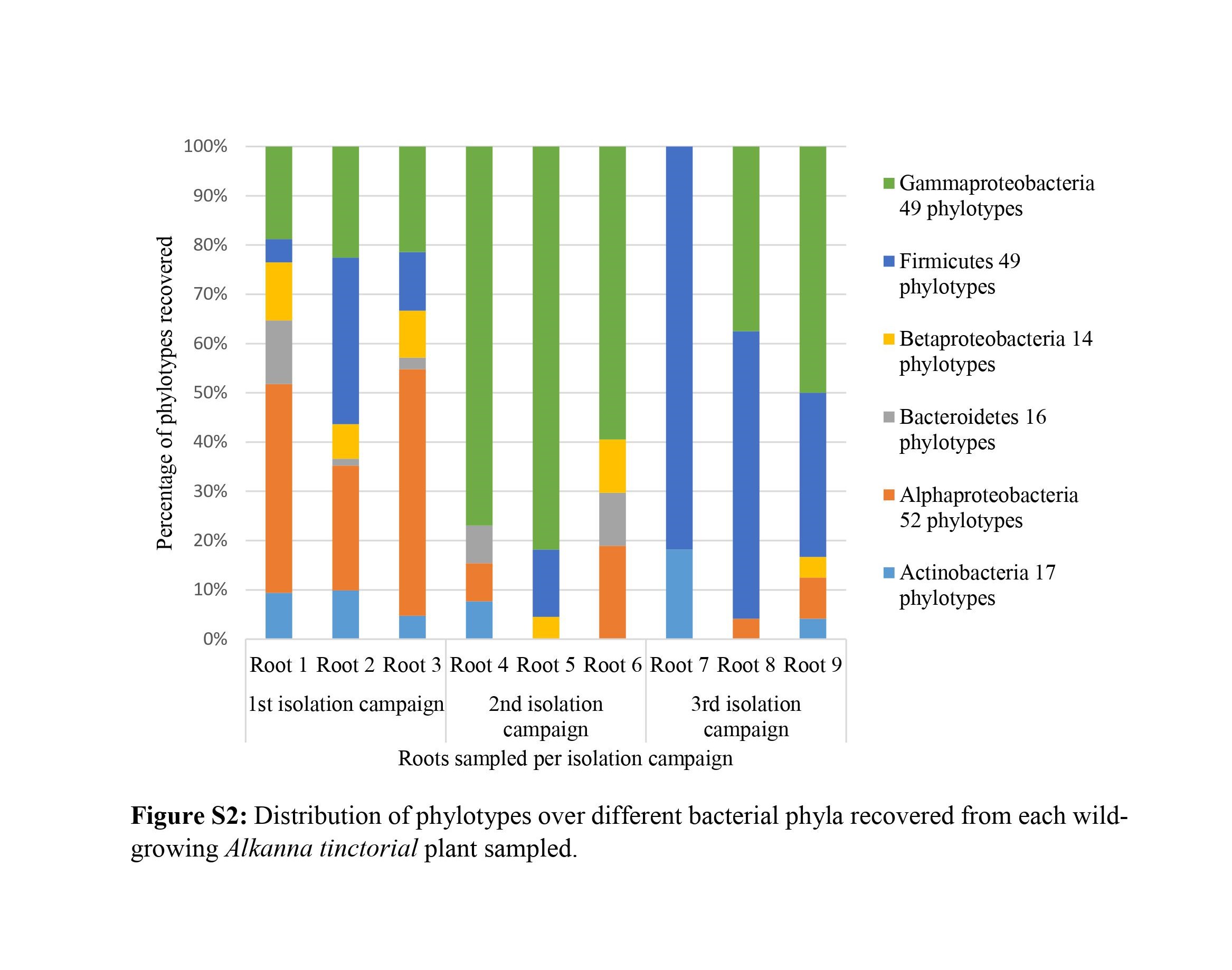

Supplement: Supplementary file 2 [file Image_2.JPEG]
