# Supplementary material for: Endophytic Bacteria From the Roots of the Medicinal Plant Alkanna tinctoria Tausch (Boraginaceae): Exploration of Plant Growth Promoting Properties and Potential Role in the Production of Plant Secondary Metabolites
Source: Front Microbiol. 2021 Feb 3;12:633488. doi: 10.3389/fmicb.2021.633488 (PMC7901983; doi:10.3389/fmicb.2021.633488)
Supplement: Supplementary file 3 [file Table_1.DOCX]

Supplementary Material

**Supplementary Table S1:** Number of bacterial isolates and phylotypes recovered from the roots of wild *Alkanna tinctoria* in different culture media. The concentrations 0.32, 1.6, 9, 32 mM are the concentrations of allantoin added to the medium 1/10 869. PE signifies that plant extract was added to 1/10 869 medium.

|  | **1^st^ isolation campaign** | | | **2^nd^ isolation campaign** | | | **3^rd^ isolation campaign** | | | **Total number of isolates** | **Total number of phylotypes** |
| --- | --- | --- | --- | --- | --- | --- | --- | --- | --- | --- | --- |
| **Medium** | **Root 1** | **Root 2** | **Root 3** | **Root 4** | **Root 5** | **Root 6** | **Root 7** | **Root 8** | **Root 9** |  |  |
| 1/10 869 | 81 | 70 | 56 | 44 | 73 | 64 | 18 | 73 | 54 | 533 | 82 |
| 1/10 869 + 0,32mM | 57 | 51 | 43 | - | - | - | - | - | - | 151 | 42 |
| 1/10 869 + 1,6mM | 71 | 64 | 56 | - | - | - | - | - | - | 191 | 40 |
| 1/10 869 + 9mM | 62 | 51 | 51 | - | - | - | - | - | - | 164 | 46 |
| 1/10 869 + 32mM | 78 | 64 | 54 | 36 | 56 | 75 | 16 | 76 | 54 | 509 | 89 |
| 1/10 869 + PE | 10 | 7 | 0 | - | - | - | - | - | - | 17 | 13 |
| ISP2 | 42 | 51 | 38 | - | - | - | - | - | - | 131 | 30 |
| 1/10 TSA | 73 | 66 | 65 | 41 | 63 | 54 | 11 | 60 | 55 | 488 | 85 |
| R2A | 86 | 72 | 64 | 75 | 98 | 91 | 15 | 80 | 70 | 651 | 81 |
| Total number of isolates | 560 | 496 | 427 | 196 | 290 | 284 | 60 | 289 | 233 | 2835 |  |
| Total number of phylotypes | 85 | 71 | 42 | 13 | 21 | 37 | 22 | 24 | 24 |  |  |

**Supplementary Table S2:** Plant growth promoting potential of the selected bacteria as tested *in vitro*. NG: no growth was observed for these bacteria; NT: Not tested; an OD ≥0,05 is positive activity for ACC deaminase; var, the biological replicates gave different results.

| **Strain** | **Name** | **Phosphate solubilisation (halo diameter in mm)** | **Siderophore production**  **(halo diameter in mm)** | **IAA production**  **(concentration in µg/mL)** | **ACC deaminase activity** | **Cellulase** | **Ligninase** | **Pectinase** | **Susceptibility to A/S** |
| --- | --- | --- | --- | --- | --- | --- | --- | --- | --- |
| **Actinobacteria** | | | | | | | | | |
| R-72562 | *Kocuria sp.* | 0 | 0 | 1.317 ±0,29 | - | - | + | - | Sensitive |
| R-72113 | *Microbacterium sp.* | 0 | 0 | 0 | - | - | - | + | Sensitive |
| R-75348 | *Micromonospora sp.* | 0 | 0 | NG | NT | NG | NG | NG | Sensitive |
| R-74611 | *Paenarthrobacter sp.* | 0 | 0 | 0,586 ±0,21 | NT | - | + | - | Sensitive |
| R-72106 | *Plantibacter sp.* | 0 | NG | 2.965 ±0.24 | - | - | - | - | Sensitive |
| R-71941 | *Tsukamurella sp.* | 0 | 2.98 | 2.734 ±0,22 | - | - | - | - | Sensitive |
| **Alphaproteobacteria** | | | | | | | | | |
| R-72379 | *Allorhizobium sp.* | 0 | 0 | 14.50 ±0,03 | var | - | + | - | Resistant |
| R-73074 | *Bradyrhizobium sp.* | 0 | 0 | 0 | - | - | - | - | Resistant |
| R-72097 | *Caulobacter sp.* | 8,99 | 3,73 | 1.198 ±0,17 | - | - | - | - | Resistant |
| R-72364 | *Caulobacter sp.* | 0 | NG | 2.709 ±0,09 | - | - | - | - | Resistant |
| R-71837 | *Ciceribacter sp.* | 2.50 | 0 | 16,45 ±0,34 | - | - | - | - | Resistant |
| R-72501 | *Inquilinus sp.* | 5,47 | 23,2 | 2.196 ±0,45 | - | - | - | - | Resistant |
| R-72380 | *Labrys sp.* | 3.56 | 0 | 2.785 ±0,42 | - | - | + | - | Resistant |
| R-72139 | *Methylobacterium sp.* | 0 | 0 | 0.629 ±0,02 | - | - | + | - | Resistant |
| R-72369 | *Methylopila sp.* | 0 | 0 | 0.733 ±0,03 | - | - | + | - | Resistant |
| R-72208 | *Neorhizobium sp.* | 0 | 0 | 6.559 ±0,70 | - | - | - | - | Resistant |
| R-72587 | *Neorhizobium sp.* | 0 | NG | 7,839 ±0,41 | - | - | - | - | Resistant |
| R-72171 | *Novosphingobium sp.* | 0 | 0 | 2.413 ±0,16 | - | - | - | + | Resistant |
| R-72085 | *Paradevosia shaoguanensis* | NG | 0 | 0 | - | - | - | + | Resistant |
| R-73111 | *Phyllobacterium sp.* | 0 | 1,87 | 6.239 ±0,73 | - | - | - | - | Resistant |
| R-71903 | *Rhizobium sp.* | 0 | 0 | 6,925 ±0,38 | - | + | - | - | Resistant |
| R-71954 | *Rhizobium alamii* | 0 | 0 | 7.605 ±0,70 | - | - | - | + | Resistant |
| R-72066 | *Rhizobium sp.* | 0 | 0 | 7.527 ±0,45 | - | + | - | - | Resistant |
| R-72160 | *Rhizobium sp.* | 0 | 0 | 19.33 ±0,24 | - | - | + | - | Resistant |
| R-72225 | *Rhizobium sp.* | 0 | 0 | 14.84 ±0,16 | - | + | - | + | Reistant |
| R-72337 | *Rhizobium wenxiniae* | 0 | 3.10 | 2.077 ±0,21 | - | - | - | + | Resistant |
| R-72433 | *Rhizobium sp.* | 0 | 1.86 | 23.56 ±0.87 | - | - | + | - | Resistant |
| R-72456 | *Rhizobium sp.* | 0 | 0 | 4,184 ±0,23 | - | - | - | - | Resistant |
| R-72475 | *Rhizobium nepotum* | 0 | NG | 9,766 ±0,29 | - | - | + | - | Resistant |
| R-72500 | *Rhizobium sp.* | 0 | 1,20 | 7,863 ±0,12 | - | - | - | - | Resistant |
| R-72553 | *Rhizobium laguerrae* | 0 | 0 | 4.197 ±0,09 | - | NG | NG | NG | Resistant |
| R-73117 | *Rhizobium skierniewicense* | 0 | 28,1 | 8,564 ±0,64 | - | - | + | - | Resistant |
| R-74576 | *Rhizobium sp.* | 2 | 2,10 | 4,922 ±0,09 | - | - | + | - | Resistant |
| R-72157 | *Rhizorhapis sp.* | 0 | NG | 0.858 ±0,08 | - | + | + | - | Resistant |
| R-71825 | *Roseomonas sp.* | 0 | 0 | 7.214±1,30 | - | - | + | - | Resistant |
| R-73070 | *Roseomonas sp.* | NG | NG | 0 | - | - | - | - | Resistant |
| R-72115 | *Shinella sp.* | 0 | 0 | 7.008 ±0,31 | - | - | - | + | Resistant |
| R-72401 | *Shinella sp.* | 0 | NG | 5.711 ±0,39 | - | + | - | - | Resistant |
| R-72459 | *Sphingobium sp.* | 0 | NG | 0,817 ±0,01 | - | - | - | - | Resistant |
| R-72591 | *Sphingobium sp.* | 0 | NG | 25.84 ±0,57 | - | - | + | - | Resistant |
| R-72354 | *Sphingomonas sp.* | 0 | 0 | 2.091 ±0,43 | var | - | - | - | Resistant |
| R-73061 | *Sphingomonas sp.* | 0 | NG | 0 | - | NG | NG | NG | Resistant |
| R-74487 | *Sphingomonas sp.* | 0 | 0 | 1,445 ±0,18 | - | - | - | - | Resistant |
| R-72597 | *Tardiphaga robiniae* | 4,98 | 17,7 | 1.946 ±0,25 | - | - | - | + | Resistant |
| **Bacteroidetes** | | | | | | | | | |
| R-72269 | *Chitinophaga ginsengisegetis* | 0 | 0 | 1.313 ±0,15 | - | - | - | - | Resistant |
| R-73072 | *Chitinophaga sp.* | 0 | NG | 0 | - | - | - | + | Resistant |
| R-72149 | *Filimonas sp.* | 0 | NG | 4.381 ±0,43 | - | - | - | + | Resistant |
| R-72247 | *Flavobacterium sp.* | 0 | 0 | 10.37 ±0,43 | - | - | - | + | Resistant |
| R-74482 | *Flavobacterium sp.* | 0 | 0 | 1,573 ±0,22 | - | - | - | + | Resistant |
| R-74536 | *Flavobacterium sp.* | 0 | 0 | 2,033 ±0,05 | - | - | - | + | Resistant |
| R-72039 | *Mucilaginibacter sp.* | 0 | 0 | 4.058 ±0,59 | var | - | - | + | Resistant |
| R-72087 | *Pedobacter sp.* | 0 | NG | 2,271 ±0,06 | - | - | - | + | Resistant |
| R-72088 | *Pedobacter sp.* | 0 | 0 | 0 | - | - | + | - | Resistant |
| R-72249 | *Pedobacter borealis* | 0 | 0 | 4.061 ±0,33 | - | - | + | + | Resistant |
| R-74587 | *Pedobacter sp.* | 0 | 2,70 | 1,441 ±0,16 | - | - | - | + | Resistant |
| R-72191 | *Olivibacter soli* | 0 | 3.40 | 1.057 ±0,006 | - | - | + | + | Resistant |
| **Betaproteobacteria** | | | | | | | | | |
| R-72367 | *Achromobacter sp.* | 0 | 1.14 | 0.073 ±0,26 | - | - | + | - | Resistant |
| R-73343 | *Acidovorax sp.* | 0 | 0 | 7,508 ±0,21 | - | - | - | - | Resistant |
| R-72091 | *Bordetella sp.* | 8.23 | 33.3 | 1.355±0,39 | - | - | + | - | Resistant |
| R-72544 | *Bordetella sp.* | 0 | 0 | 3,293 ±0,13 | - | - | + | - | Resistant |
| R-73148 | *Duganella sp.* | 0 | 0 | 6.959 ±0,63 | - | NG | NG | NG | Resistant |
| R-72395 | *Massilia sp.* | 0 | 0 | 4.697 ±0,05 | - | - | - | + | Resistant |
| R-72419 | *Pigmentiphaga aceris* | 2.83 | 0 | 7.921 ±0,27 | - | - | - | - | Resistant |
| R-72060 | *Variovorax ginsengisoli* | 0 | 0 | 0.110 ±0,08 | NT | + | - | - | Resistant |
| R-72153 | *Variovorax ginsengisoli* | 0 | 0 | 0.104 ±0,04 | - | + | - | - | Resistant |
| R-72446 | *Variovorax sp.* | 0 | 0 | 3,212 ±0,27 | - | - | - | - | Resistant |
| R-72495 | *Variovorax sp.* | 0 | 0 | 6.968 ±0,15 | + | - | + | - | Resistant |
| **Firmicutes** | | | | | | | | | |
| R-71875 | *Brevibacteriumsp.* | 0 | 0 | 0 | - | - | - | - | Sensitive |
| R-71893 | *Bacillus sp.* | 2.31 | 0 | 4.042 ±0,76 | - | - | - | - | Sensitive |
| R-71922 | *Bacillus sp.* | 0 | 0 | 3,038 ±0,14 | - | - | + | - | Sensitive |
| R-72189 | *Bacillus sp.* | 0 | 0 | 11.06 ±0,15 | - | - | + | - | Sensitive |
| R-72492 | *Bacillus sp.* | 0 | 0 | 0 | - | - | + | - | Sensitive |
| R-72633 | *Bacillus sp.* | 0 | 0 | 1.414 ±0,34 | + | - | - | - | Sensitive |
| R-72634 | *Bacillus sp.* | 0 | 13,6 | 1,838 ±0,08 | - | - | + | + | Sensitive |
| R-74209 | *Bacillus sp.* | 0 | 0 | 0 | - | - | - | - | Sensitive |
| R-74277 | *Bacillus solisilvae* | 0 | 0 | 0,733 ±0,04 | - | - | + | + | Sensitive |
| R-74294 | *Bacillus sp.* | 3,84 | 4,84 | 8.511 ±0.59 | + | - | + | - | Sensitive |
| R-71830 | *Brevibacillus sp.* | 0 | NG | 5.464 ±0,30 | - | + | - | + | Sensitive |
| R-71971 | *Brevibacillus sp.* | 0 | NG | 6.795 ±0.69 | NT | - | - | - | Sensitive |
| R-74223 | *Brevibacillus borstelensis* | 0 | 0 | 7,292 ±0.16 | - | - | - | - | Sensitive |
| R-74266 | *Brevibacillus formosus* | 0 | 0 | 6,391 ±0,48 | var | - | - | - | Sensitive |
| R-73096 | *Cohnella sp.* | NG | NG | 2.322 ±0,16 | - | NG | NG | NG | Sensitive |
| R-72005 | *Paenibacillus xylanisolvens* | NG | NG | 2.039 ±0,16 | - | - | - | - | Sensitive |
| R-74146 | *Paenibacillus chitinolyticus* | 0 | 0 | 2,419 ±0,12 | - | - | - | - | Sensitive |
| R-74291 | *Paenibacillus lautus* | 0 | NG | 0,542 ±0,03 | var | - | - | - | Sensitive |
| R-73089 | *Staphylococcus sp.* | 0 | 0 | 0 | var | - | - | - | Sensitive |
| R-73090 | *Staphylococcus warneri* | 0 | 0 | 0 | - | - | - | - | Sensitive |
| **Gammaproteobacteria** | | | | | | | | | |
| R-71889 | *Acinetobacter johnsonii* | 0 | 0 | 5.491 ±0,23 | - | - | + | - | Resistant |
| R-74161 | *Acinetobacter sp.* | 0 | 0 | 14,07 ±0,78 | - | - | + | - | Resistant |
| R-74597 | *Acinetobacter sp.* | 2,93 | 3,06 | 2,748 ±0,41 | - | - | + | - | Resistant |
| R-72417 | *Enhydrobacter sp.* | 0 | NG | 1,378 ±0,06 | - | - | - | - | Resistant |
| R-72151 | *Luteibacter sp.* | 0 | 15.9 | 1.832 ±0,15 | - | - | + | - | Resistant |
| R-73110 | *Luteibacter sp.* | 0 | 5,85 | 2.161 ±0,22 | - | - | - | - | Resistant |
| R-72015 | *Pantoea sp.* | 3.64 | 29.3 | 10.24 ±1,84 | - | - | + | - | Resistant |
| R-72163 | *Pantoea sp.* | 5.21 | 26.8 | 9.992 ±0.29 | - | - | - | - | Resistant |
| R-72394 | *Pantoea sp.* | 5.39 | 22.5 | 4.062 ±0,44 | - | - | + | + | Resistant |
| R-72498 | *Pantoea sp.* | 4,15 | 21,2 | 9,696 ±0,08 | - | - | + | - | Resistant |
| R-74258 | *Pantoea sp.* | 5,03 | 7,34 | 8,830 ±0,22 | - | - | + | - | Resistant |
| R-74476 | *Pantoea sp.* | 4,10 | 6,55 | 9.252 ±0.12 | - | - | + | - | Resistant |
| R-71838 | *Pseusomonas sp.* | 5.06 | 33.1 | 11,65 ±0,14 | - | - | + | - | Resistant |
| R-71842 | *Pseusomonas sp.* | 5.09 | 29.5 | 8.258 ±0.63 | - | - | - | - | Resistant |
| R-71976 | *Pseudomonas sp.* | 4.47 | 2.23 | 13.03±0.40 | NT | - | + | - | Resistant |
| R-71997 | *Pseudomonas sp.* | 5.84 | 28.3 | 24.48 ±0,70 | - | - | + | - | Resistant |
| R-72008 | *Pseudomonas sp.* | 6.98 | 19.3 | 7.816 ±0,35 | + | - | + | - | Resistant |
| R-72074 | *Pseudomonas sp.* | 4.94 | 18.8 | 35.5 ± 1,61 | - | - | + | - | Resistant |
| R-72102 | *Pseudomonas sp.* | 5.58 | 34.43 | 15.49 ± 0,24 | - | - | - | - | Resistant |
| R-72135 | *Pseudomonas sp.* | 6.35 | 15.2 | 36.88 ±0,37 | + | - | + | - | Resistant |
| R-72164 | *Pseudomonas sp.* | 3,56 | 22,8 | 27.4 ±1.3 | - | - | + | - | Resistant |
| R-72172 | *Pseudomonas sp.* | 4.89 | 35.1 | 13.96 ±0.80 | - | - | + | - | Resistant |
| R-72210 | *Pseudomonas sp.* | 4.52 | 1.23 | 5.588 ±0,58 | - | - | + | + | Resistant |
| R-72599 | *Pseudomonas turukhanskensis* | 0 | 40,8 | 15,25 ±084 | - | - | - | - | Resistant |
| R-74216 | *Pseudomonas sp.* | 3,73 | 24,9 | 22,03 ±0,45 | - | - | - | - | Resistant |
| R-74322 | *Pseudomonas sp.* | 4,89 | 32,7 | 24,53 ±0,53 | - | - | + | - | Resistant |
| R-74327 | *Pseudomonas sp.* | 3,96 | 26,6 | 24,14 ±0,58 | + | - | + | - | Resistant |
| R-74442 | *Pseudomonas sp.* | 6,7 | 14,5 | 12,42 ±0,66 | - | - | - | - | Resistant |
| R-74520 | *Pseudomonas sp.* | 2,81 | 3,22 | 8,278 ±0,19 | - | - | - | - | Resistant |
| R-74612 | *Pseudomonas sp.* | 5,79 | 7,24 | 13.09 ±0,61 | - | - | + | - | Resistant |
| R-71986 | *Pseudoxanthomonas spadix* | 0 | NG | 4.745 ±0.24 | - | - | - | - | Resistant |
| R-72406 | *Stenotrophomonas sp.* | 0 | 1.73 | 5,042 ±0,35 | - | - | + | - | Resistant |
| R-74210 | *Stenotrophomonas sp.* | 0 | 4,05 | 3.855 ±0,20 | - | - | + | - | Resistant |
| R-74235 | *Stenotrophomonas sp.* | 0 | 3,61 | 3.684 ±0,16 | - | - | + | - | Resistant |
| R-74283 | *Stenotrophomonas sp.* | 0 | 3,72 | 4,774 ±0,32 | - | - | + | - | Resistant |
| R-74546 | *Stenotrophomonas sp.* | 0 | 3,49 | 7,189 ±0,16 | - | - | + | - | Resistant |
| R-74547 | *Stenotrophomonas sp.* | 0 | 2,60 | 4,310 ±0,14 | - | - | + | - | Resistant |
| R-72251 | *Xanthomonas sp.* | 0 | 1.88 | 2,879 ±0,40 | NT | - | - | + | Resistant |
| R-72464 | *Xanthomonas sp.* | 0 | 0 | 8,470 ±0,44 | - | - | + | + | Resistant |
| R-73098 | *Xanthomonas sp.* | 0 | 2.28 | 2.879 ±0,40 | - | - | - | + | Resistant |

Supplementary Table S3: GenBank accession numbers for the partial 16S rRNA gene sequences of 127 strains tested for plant-growth promoting activities, enzymatic activities and susceptibility to alkannin and shikonin.

| **Strain** | **Accession number** | **Strain** | **Accession number** | **Strain** | **Accession number** |
| --- | --- | --- | --- | --- | --- |
| R-71825 | MW353471 | R-72151 | MW353506 | R-72597 | MW353552 |
| R-71830 | MW353472 | R-72153 | MW353507 | R-72599 | MW353553 |
| R-71837 | MW353473 | R-72157 | MW353508 | R-72633 | MW353554 |
| R-71838 | MW353474 | R-72160 | MW353509 | R-72634 | MW353555 |
| R-71842 | MW353475 | R-72163 | MW353510 | R-73061 | MW353556 |
| R-71875 | MW353476 | R-72164 | MW353511 | R-73070 | MW353557 |
| R-71889 | MW353477 | R-72171 | MW353512 | R-73072 | MW353558 |
| R-71893 | MW353478 | R-72172 | MW353513 | R-73074 | MW353559 |
| R-71903 | MW353479 | R-72189 | MW353514 | R-73089 | MW353560 |
| R-71922 | MW353480 | R-72191 | MW353515 | R-73090 | MW353561 |
| R-71941 | MW353481 | R-72208 | MW353516 | R-73096 | MW353562 |
| R-71954 | MW353482 | R-72210 | MW353517 | R-73098 | MW353563 |
| R-71971 | MW353483 | R-72225 | MW353518 | R-73110 | MW353564 |
| R-71976 | MW353484 | R-72247 | MW353519 | R-73111 | MW353565 |
| R-71986 | MW353485 | R-72249 | MW353520 | R-73117 | MW353566 |
| R-71997 | MW353486 | R-72251 | MW353521 | R-73148 | MW353567 |
| R-72005 | MW353487 | R-72269 | MW353522 | R-73343 | MW353568 |
| R-72008 | MW353488 | R-72337 | MW353523 | R-74146 | MW353569 |
| R-72015 | MW353489 | R-72354 | MW353524 | R-74161 | MW353570 |
| R-72039 | MW353490 | R-72364 | MW353525 | R-74209 | MW353571 |
| R-72060 | MW353491 | R-72367 | MW353526 | R-74210 | MW353572 |
| R-72066 | MW353492 | R-72369 | MW353527 | R-74216 | MW353573 |
| R-72074 | MW353493 | R-72379 | MW353528 | R-74223 | MW353574 |
| R-72085 | MW353494 | R-72380 | MW353529 | R-74235 | MW353575 |
| R-72087 | MW353495 | R-72394 | MW353530 | R-74258 | MW353576 |
| R-72088 | MW353496 | R-72395 | MW353531 | R-74266 | MW353577 |
| R-72091 | MW353497 | R-72401 | MW353532 | R-74277 | MW353578 |
| R-72097 | MW353498 | R-72406 | MW353533 | R-74283 | MW353579 |
| R-72102 | MW353499 | R-72417 | MW353534 | R-74291 | MW353580 |
| R-72106 | MW353500 | R-72419 | MW353535 | R-74294 | MW353581 |
| R-72113 | MW353501 | R-72433 | MW353536 | R-74322 | MW353582 |
| R-72115 | MW353502 | R-72446 | MW353537 | R-74327 | MW353583 |
| R-72135 | MW353503 | R-72456 | MW353538 | R-74442 | MW353584 |
| R-72139 | MW353504 | R-72459 | MW353539 | R-74476 | MW353585 |
| R-72149 | MW353505 | R-72464 | MW353540 | R-74482 | MW353586 |
| R-72151 | MW353506 | R-72475 | MW353541 | R-74487 | MW353587 |
| R-72153 | MW353507 | R-72492 | MW353542 | R-74520 | MW353588 |
| R-72157 | MW353508 | R-72495 | MW353543 | R-74536 | MW353589 |
| R-72160 | MW353509 | R-72498 | MW353544 | R-74546 | MW353590 |
| R-72163 | MW353510 | R-72500 | MW353545 | R-74547 | MW353591 |
| R-72164 | MW353511 | R-72501 | MW353546 | R-74576 | MW353592 |
| R-72171 | MW353512 | R-72544 | MW353547 | R-74587 | MW353593 |
| R-72172 | MW353513 | R-72553 | MW353548 | R-74597 | MW353594 |
| R-72135 | MW353503 | R-72562 | MW353549 | R-74611 | MW353595 |
| R-72139 | MW353504 | R-72587 | MW353550 | R-74612 | MW353596 |
| R-72149 | MW353505 | R-72591 | MW353551 | R-75348 | MW353597 |
